# Socioeconomic inequalities in depression and the role of job conditions in China

**DOI:** 10.3389/fpubh.2024.1464187

**Published:** 2024-12-12

**Authors:** Jie Ma, Jinjing Wu, Guillaume Marois

**Affiliations:** ^1^Asian Demographic Research Institute, School of Sociology and Political Sciences, Shanghai University, Shanghai, China; ^2^International Institute for Applied Systems Analysis, Wittgenstein Centre for Demography and Global Human Capital (IIASA, OeAW), University of Vienna, Laxenburg, Austria

**Keywords:** depression, socioeconomic status (SES), job conditions, working hours, stress of higher status

## Abstract

**Background:**

The rising prevalence of depression in China, coupled with a tightening job market, highlights concern for the workforce’s mental health. Although socioeconomic inequalities in depression have been well documented in high-income countries, the association between socioeconomic status (SES) and depression, along with its work-related mediators, has not been sufficiently studied in China.

**Methods:**

The study participants are 6,536 non-agriculturally employed working adults from the 2020 China Family Panel Studies (CFPS). We build linear regression models to examine the relationship between SES and depression, using education and income as indicators of SES. We also apply a framework based on seemingly unrelated estimation (SUEST) to assess how job conditions, which include job demands and job resources, mediate this relationship.

**Results:**

Both education and income are negatively associated with depression, with education’s association with depression remaining net of income. Mediation analysis reveals that the well-educated tend to occupy less demanding work with shorter working hours and lower probability of on-call duty, which partially helps explain the education-based depression gap. Higher earners experience more demanding work with longer working hours and higher probability of on-call duty, which potentially masks the income-based depression gap. Greater job resources including moderate schedule flexibility and better job security, appear to contribute to explaining the depression gap across SES.

**Limitation:**

The cross-sectional design of this study precludes causal inferences. Not all typical job demands and resources could be included due to data limitations.

**Conclusion:**

Our study provides insights into socioeconomic inequalities in mental health in the Chinese working population, with implications for policies aimed at preventing depression and improving mental health equity.

## 1 Introduction

Mental disorders rose to the sixth leading cause of global health loss in 2020, up from tenth in 2010, indicating their increasing impact on global health (1). Depression, one of the most common mental disorders, affected 325 million people worldwide, accounting for approximately 4.3% of the global population (1). Depression is often a recurrent and lifelong illness that impairs psychosocial functioning and reduces quality of life (2, 3). In 2020, it accounted for the largest share (36.4%) of disability-adjusted life years attributable to mental disorders (1).

The global employment-to-population ratio is approximately 60% (4), highlighting the significance of addressing depression in the working population. The prevalence of mental illness is notably high among the working population, with 15 percent of working-age adults experiencing mental disorder (5), which can reduce healthy life expectancy and productivity, with substantial health, social, and health consequences (6–8). Effectively preventing depression among workers not only improves their quality of life but also boosts economic productivity and enhances the well-being of their dependents.

Growing research attention on the social determinants of depression highlights the role of socioeconomic status (SES), often measured through education or income levels, as a key factor (9–11). Despite extensive research from high-income countries, low- and middle-income countries, which experience over 80% of the health loss from depression and face pronounced socioeconomic inequalities, remain insufficiently explored (12, 13).

China, a middle-income country, has experienced rapid economic growth accompanied by increasing income inequality (14). This context has fostered “involution (neijuan),” or intense internal competition (15), and a culture of overwork (16), contributing to the emergence of depression as a significant public health concern. In 2020, 51 million adults aged 20 and older in China were suffering from depressive disorders, representing 17.3% of global cases (1). The examination of socioeconomic inequalities in depression within China’s working population could enhance global discourse on this topic and offer insights for other emerging economies with similar economic dynamics and cultural backgrounds.

Identifying the mediators that link SES to depression is essential for a deeper understanding of the mechanisms underlying socioeconomic inequalities in depression. For the working population, job conditions may serve as a crucial mediator in the association between SES and depression. Drawing on the Job Demand-Resource model, job conditions can be categorized into job demands (also referred to as stressors) and job resources, offering a framework for understanding their impact (17).

Job demands, such as long working hours, irregular work schedules, and work-family boundary-crossing requirements, are challenging aspects of work that may lead to depression (17). Some Western studies suggest a “stress of higher status” pattern, in which increased job demands among higher SES individuals mask socioeconomic disparities in depression (18, 19). These concerns are rooted in Coser’s “greedy institution” theory (20) and Blair-Loy’s concept of the “work devotion schema” (21), which posits that higher-status workers face limitless demands for effort and energy due to the intense allegiance and unwavering commitment required of them (22). Conversely, other research indicates that lower SES individuals face heavier job demands, contributing to socioeconomic inequalities in depression, which suggest a “stress of lower status” pattern (23–25). While there is a lack of evidence from non-Western contexts to contribute to this debate, it is possible that higher SES individuals in China may experience increased depression due to the stressors associated with maintaining or improving their status in a highly competitive labor market, although further investigation is needed.

Job resources include beneficial aspects such as schedule control, job authority, and job security (26). It is hypothesized that an abundance of job resources that serve as a sense of mastery and perceived control among those with higher SES may contribute to their lower depression levels (27, 28), but empirical examination of job resources as a mediator in the SES-depression relationship has been limited, particularly in China.

It is crucial to separately investigate how income and education correlate with depression to fully understand how depression is distributed across SES. Each measure—education and income—captures a unique aspect of SES, potentially linking to depression through different mechanisms (12, 29, 30), and their impact can vary significantly across societies (31–34). Given the cultural emphasis on academic success in China (35, 36), we specifically investigate whether education is associated with lower depression levels, independent of income. Furthermore, a detailed examination of how job demands and resources mediate the relationships between education, income, and depression could reveal distinct pathways associated with depression. This analysis would complement existing research that has identified various patterns in how job conditions mediate these relationships (11).

In sum, the present study attempts to fill the gaps by investigating the following questions: For China’s working population, (1) What is the relationship between SES and depression? Both income and education are considered as SES indicators to obtain a complete picture from different dimensions of this relationship. (2) How do job conditions, divided into job demands and job resources, mediate the education- and income-depression relationship? We analyze the potential mediating effect of job conditions in the relationship between SES and depression to test whether the Chinese evidence favors the “stress of higher status” pattern or suggests an alternative explanation.

## 2 Data and methods

### 2.1 Sample

**Figure 1:**
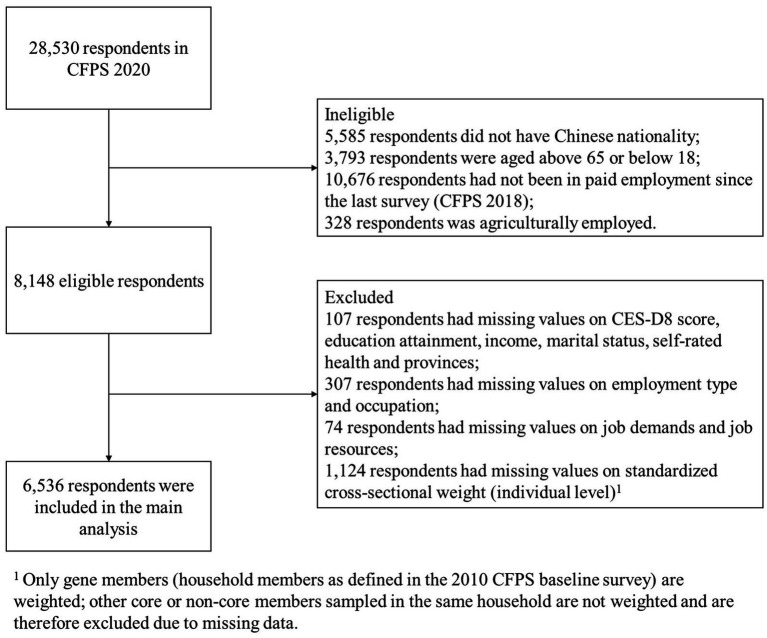
Sample selection flow chart.

The data is derived from the China Family Panel Studies (CFPS), a nationally representative, biennial longitudinal survey conducted by the Institute of Social Science Survey (ISSS) at Peking University since 2010. The survey collects a wide range of variables at the individual, family, and community levels, aiming to reflect the changes in China society, economy, education, and health. The CFPS sample covers 25 provinces, cities, and autonomous regions, representing approximately 95% of China’s population. It employs a multi-stage, multi-level probability proportional to size (PPS) sampling method, with a three-stage sampling of “district/county-village/neighborhood committee-household.” In the present study, we used survey data collected in 2020, with a cross-sectional response rate at the household level of 62%. We selected non-agriculturally employed workers between the ages of 18 and 65, and removed cases with missing values, leaving a final sample of *N* = 6,536. The sample selection process is shown in Figure 1. To ensure that the 2020 wave maintains its representativeness of China’s population, CFPS applies cross-sectional weights to adjust for sample attrition and enhance alignment with national demographic structures.

### 2.2 Variables

#### 2.2.1 Depression measure

The Center for Epidemiologic Studies Depression (CES-D8) scale, created by Radloff (37), was utilized in the 2020 CFPS survey to measure depression. Respondents reported their frequency of eight negative feelings experienced in the past week, such as feeling lonely or sad, having trouble sleeping, or finding it difficult to enjoy life. Response options range from “never (less than one day),” “sometimes (1–2 days),” “often (3–4 days),” to “most of the time (5–7 days),” with scores ranging from 0 to 3. The total score on the scale ranges from 0 to 24, with higher scores indicating more severe depressive symptoms. The CES-D-8 scale has been shown to have good reliability and validity in screening for depressive symptoms in previous studies (38, 39).

#### 2.2.2 SES measures

One of the authors of the “stress of higher status” hypothesis, used both education and occupational income as indicators of occupational SES (11, 29) to test the hypothesis. We follow his lead to investigate the SES-depression relationship, as well as how job conditions mediate it.

*Education* is categorized into 4 levels ranging from “less than high school,” “high school and technical secondary school,” “3-year vocational college,” to “4-year college and above.”

*Occupational income* is defined as total income from all paid employment in the past 12 months and is compared by quintile in our analyses.

##### 2.2.2.1 Job conditions measures

Unlike other popular psychological models in the occupational context, the Job Demands-Resources (JD-R) model encompasses a broader range of job characteristics, integrating elements from other models and assuming any demand or resource can impact employees’ health and well-being (40, 41). Hence, we choose the JD-R model for theoretical analyses to examine how the job stressors and resources are related to depression and in which way they mediate the SES-depression association. The JD-R model includes various indicators for job demands and resources. While the China Family Panel Studies (CFPS) offers limited job-related variables, it provides the best coverage and response rate among available public datasets in China, to our knowledge. Therefore, we utilize CFPS job-related variables as proxies for job demands and resources.

##### 2.2.2.2 Job demands measures

The “stress of higher status” hypothesis suggests that higher-SES individuals face greater job demands, particularly longer working hours and work-family role blurring and conflict (19, 28), which may contribute to increased mental illness such as anxiety and depression (11, 40, 42). The frequency of work-related contact outside of normal working hours has been employed in some studies to measure the extent of work-family role blurring (11, 43). Similarly, on-call duty in our study, which requires around-the-clock contact and work availability, fosters the permeability of work-nonwork boundaries (44, 45), and thus can also be taken as a proxy for role blurring. Studies suggest that on-call duty causes mental illness, including depression (46). However, some other studies suggest an opposite pattern of “stress of lower status” with evidence that low-status workers tend to work longer hours and are more likely to be on-call (23–25). Shift work (i.e., night shift, weekend shift), which represents the demands of work abnormally scheduled outside standard daytime, has also been found to be associated with lower status (47), and serves as a significant predictor of depression (8, 48).

Here, we measure *weekly working hours* based on the average hours per week the respondent worked for the job in the past 12 months. We measure *on-call duty* by the item “Is it because of the demands of the job that your phone cannot be switched off 24 h a day and you are always on call?.” Individuals who answered yes are coded 1, while others are coded 0. *Nightshift frequency* is measured by one’s average frequency of working night shift in the past 12 months. Responses are categorized as: “never” (the reference group), “no more than once a week,” and “several times a week/every day.” *Weekend at work frequency* is measured by the average frequency of working on weekends in the past 12 months. Responses are categorized as: “no more than once a month” (the reference group), “several times a month but less than every week,” and “every week.”

##### 2.2.2.3 Job resources measures

Job resources refer to aspects of job that should help workers manage job pressure, including schedule flexibility, job authority and job security (26). These resources are generally found to be closely related to higher statuses (49, 50) and lower depression (22, 51, 52), with the exception of the relationship between job authority and mental health, which has reached mixed conclusions (53, 54).

*Schedule flexibility*, representing employees’ discretion over the timing and duration of work (55), is measured by the item “How flexible have you been with your working hours for this job?.” Here, working hours refer to when you start and finish work, not the total number of hours you work each week. Responses are categorized as: “(Completely flexible) The working hours are not fixed. They depend on work needs, and determined by myself,” “(Semi-flexible) There are fixed hours and I can adapt the hours with certain limits” (the reference group), and “(Completely fixed) They are completely fixed or set by the superintendent.” Regarding job authority, involving control over others’ work (55), direct supervision is widely taken as an adequate proxy for measuring the authority resource in the absence of a detailed subjective indicator of job authority (56–58). We measure *direct supervision* by the item “Do you have any direct subordinates?.” Individuals who answered yes are coded 1, while others are coded 0. *Job security*, which can be considered a perceived organizational resource leading to work stability and durability (26, 40), is measured by the item “How satisfied are you with this job security?” Responses are scored on a five-point Likert scale ranging from “very unsatisfied” (1) to “very satisfied” (5).

#### 2.2.3 Control variables

Sociodemographic variables include *gender*, *age*, *age*^2^, *marital status*, *self-rated health* and *region*. We also control for some job-related covariates such as *employment type* and *occupation*. As for *employment type*, the public sector refers to employment in government departments, people’s organizations, and public institutions. The non-private sector refers to employment in state-owned enterprises. The private sector refers to employment in private businesses. As for *occupation*, we use the CFPS-provided codes to compare “legislators, senior officials and manager,” “professional,” “technicians,” “clerks,” “service and sales,” “trade workers,” “plant and machine operators and assemblers,” and “elementary workers.”

Table 1 provides a descriptive analysis of key variables used in the study, including measures of depression, socioeconomic status (SES), job demands, job resources and control variables.

**Table 1:** Descriptive statistics of variables included in the analysis (*N* = 6,536).^1^

Depression Measure		Job Demands Measures	
CESD8 (0-24)	5.3 (3.8)	**Weekly working hours**	53 (18)
		**On-call duty**	
**SES Measures**		Not required to be on-call	3164 (48%)
**Education Level**		Required to be on-call	3372 (52%)
Less than High school	2967 (45%)	**Nightshift frequency**	
High school and technical secondary school	1334 (20%)	Never	4247 (65%)
3-year vocational college	1099 (17%)	No more than once a week	1231 (19%)
4-year college and above	1136 (17%)	Several times a week/every day	1058 (16%)
		**Weekend at work frequency**	
**Occupational Income**^2^		No more than once a month	1635 (25%)
Income quantile 1 (20000RMB or less)	1360 (21%)	Several times a month but less than every week	983 (15%)
Income quantile 2 (20001RMB-35000RMB)	1293 (20%)	Every week	3918 (60%)
Income quantile 3 (35001RMB-50000RMB)	1587 (24%)		
Income quantile 4 (50001RMB-70000RMB)	994 (15%)	**Job Resources Measures**	
Income quantile 5 (70001RMB or more)	1302 (20%)	**Direct supervision**	
		Not holding a direct supervisory position	5520 (84%)
**Sociodemographic Characteristics**		Holding a direct supervisory position	1016 (16%)
**Age**	39 (11)	**Job security**	3.88 (0.91)
		**Schedule flexibility**	
**Urban**		Completely fixed	3518 (54%)
Rural	4150 (63%)	Completely flexible	1001 (15%)
Urban	2386 (37%)	Semi-flexible	2017 (31%)
			
**Gender**		**Other Job-Related Covariates**	
Male	3858 (59%)	**Employment type**	
Female	2678 (41%)	Public sector	1158 (18%)
		Non-private sector	906 (14%)
**Married**		Private sector	4472 (68%)
Single/Divorced/Widowed	1926 (29%)		
Married	4610 (70%)	**Occupation**	
		Legislators, senior officials and manager	364 (5.6%)
**Self-rated health (0-5)**	3.29 (1.05)	Professionals	1124 (17%)
		Technicians and associate professionals	514 (7.9%)
**Region**		Clerks	468 (7.2%)
East	3186 (49%)	Service and sales workers	1472 (23%)
Middle	1829 (28%)	Elementary workers	577 (8.8%)
West	1537 (23%)	Craft and trade workers	1228 (19%)
		Plant and machine operators and assemblers	789 (12%)

1 Mean (SD); n (%).

2 Each income quantile is not exactly equal because of tied observations at the boundaries between quantiles.

### 2.3 Statistical analyses

The analysis is divided in two main sections. In the first one, to illustrate the SES-job demands and SES-job resources gradient, we look at the distribution of job demands and job resources across different levels of education and income. Depending on the nature of the job demands or resources measures as independent variables, appropriate statistical models are employed, including OLS regression for continuous variables such as weekly working hours and job security, binary logistic regression for binary variables such as on-call duty and direct supervision, ordinal logistic regression for ordinal variables including nightshift frequency and weekend at work frequency, and multinomial logistic regression for schedule flexibility, a categorical variable with three categories.

In the second section, we use linear regression models to evaluate the hypotheses about the SES-depression relationship, and then assess the mediator roles of job demands and job resources. The baseline model (Model A) only includes the two SES indicators, education and income, and control variables. Subsequent models successively include different job demands (Models S1-S5) and job resources (Models S7-S9), as shown in Supplementary Table S2. All the above analyses are performed in R 4.3.1. All regression models are weighted by the “standardized cross-sectional weight (individual level)” to adjust for potential sampling biases and ensure representativeness of the results.

To examine the mediating effects of job demands and resources on the relationship between education, income, and depression, we apply a framework based on seemingly unrelated estimation (SUEST) (59), which facilitates the calculation of cross-model covariances to test the equality of SES coefficients across models, as well as the discrete change in the SES coefficients between the baseline and subsequent models, which helps to evaluate how job conditions might mediate the relationship between SES and depression. The above cross-model tests and calculations are performed in Stata 17.0, via the “gsem” command.

Considering family economic status may confound the SES-depression relationship, we conduct a sensitivity analysis to assess the robustness of our main findings after adjusting for household income (as shown in Model B) in a reduced sample (*N* = 6,397) due to the high rate of missing data for this variable.

## 3 Results

### 3.1 The association between SES and depression

The baseline model (Model A) in Table 2 shows that both education and income are negatively associated with depression, controlling for each other and other conditions. With the exception of the highest income group, depression decreases across SES groups. The 4-year college and above group and the second highest income group exhibit the greatest reduction in depression scores compared to the lowest groups.

**Table 2:** Regression analysis of depression on socioeconomic status (SES; *N* = 6,536).

	Model A (Baseline model)
Education level (Reference = Below high school)	
High school and technical secondary school	−0.314* (0.127)
3-year vocational college	−0.622*** (0.154)
4-year college and above	−0.752*** (0.180)
Occupational Income (Reference = Income quantile 1)	
Income quantile 2	−0.526*** (0.141)
Income quantile 3	−0.787*** (0.139)
Income quantile 4	−1.116*** (0.160)
Income quantile 5	−0.899*** (0.158)
Intercept	7.504 *** (0.729)
R^2^/Adjusted R^2^	0.103 / 0.100
AIC	38625.212

*p < 0.05, **p < 0.01, ***p < 0.001. All models control for age, age square, gender, marital status, urban residence, self-rated health, region residence, employment type and occupation. Standard errors are in parentheses. AIC stands for Akaike Information Criterion.

### 3.2 The mediating effect of job conditions

First, we examine the relationship between SES and job demands and job resources. As shown in Supplementary Table S1A, regarding the association between education and job demands, weekly working hours and weekend at work frequency decline across all education levels. Higher education levels are associated with lower nightshift frequency, though the difference between the high school and the lowest education group is not statistically significant. Additionally, the 4-year college and above group, representing the highest education level in our study, exhibits the largest and statistically significant reduction in the likelihood of on-call duty compared to the lowest education group, whereas differences between other education groups and the lowest education group are not statistically significant. In contrast, the association between income and job demands shows a different pattern from that of education. Income is positively associated with weekly working hours and on-call duty, except that the difference between income quantile 2 and the reference group (income quantile 1) is not statistically significant. Furthermore, income is not significantly associated with nightshift frequency or weekend work frequency.

As shown in Supplementary Table S1B, regarding the association between SES and job resources, individuals with higher education and income levels generally have moderate schedule flexibility (also referred to as semi-fixed flexibility) compared to those with lower education and income levels. Additionally, those with higher education and income are more likely to hold supervisory positions, although the difference between income quantile 2 and the lowest income level is not statistically significant. Higher education is also associated with greater job security, though the difference between high school graduates and the lowest education group is not statistically significant. Among income groups, only the highest earners report significantly better job security than the lowest earners, with no significant differences found between the other income levels and the lowest income level.

Next, we analyze the association between job conditions and depression. As shown in Supplementary Table S2, the analysis of job demands indicates that weekly working hours, on-call duty, nightshift frequency and weekend at work frequency are associated positively with depression. The analysis of job resources suggests that resources including moderate schedule flexibility and job security are associated negatively with depression. However, holding supervisory positions is not significantly associated with depression.

In examining the potential mediating effect of job conditions in the SES-depression association, we then compute the cross-model differences for the discrete change in the estimated coefficient of a level of SES before and after (after minus before) controlling for each measure of job demand or job resource, as shown in Tables 3, 4, and assess their statistical significance.

**Table 3:** Cross-model difference for discrete change (after-before) of a level of socioeconomic status (SES) after controlling for job demands (*N* = 6,536).

	Job demands			
	Weekly working hours	On-call duty	Nightshift frequency	Weekend at work frequency
Education level (Reference = Below high school)				
High School and Technical secondary school	−0.079** (0.027)	0.000 (0.013)	−0.008 (0.013)	0.022 (0.021)
3-year vocational college	−0.146** (0.046)	−0.018 (0.019)	−0.029 (0.019)	0.003 (0.035)
4-year college and above	−0.160** (0.052)	−0.046* (0.021)	−0.035 (0.022)	−0.003 (0.043)
Occupational income (Reference = Income quantile 1)				
Income quantile 2	0.037* (0.017)	0.003 (0.015)	−0.009 (0.013)	0.007 (0.011)
Income quantile 3	0.035* (0.017)	0.034* (0.017)	0.004 (0.014)	0.011 (0.012)
Income quantile 4	0.021 (0.016)	0.019 (0.017)	−0.012 (0.016)	0.007 (0.014)
Income quantile 5	0.042† (0.022)	0.018 (0.018)	0.012 (0.018)	0.006 (0.012)

†*p* < 0.1, *p < 0.05, **p < 0.01, ***p < 0.001. All models control for age, age square, gender, marital status, urban residence, self-rated health, region residence, employment type and occupation. Standard errors are in parentheses.

**Table 4:** Cross-model difference for discrete change (after-before) of a level of socioeconomic status (SES) after controlling for job resources (*N* = 6,536).

	Job resources		
	Schedule flexibility	Direct supervision	Job security
Education level (Reference = Below high school)			
High school and technical secondary school	−0.025 (0.015)	−0.002 (0.010)	−0.017 (0.018)
3-year vocational college	−0.030† (0.017)	−0.003 (0.016)	−0.041 (0.025)
4-year college and above	−0.036† (0.020)	−0.002 (0.011)	−0.085** (0.030)
Occupational income (Reference = Income quantile 1)			
Income quantile 2	−0.014 (0.015)	0 (0.002)	0.007 (0.019)
Income quantile 3	−0.019 (0.019)	−0.001 (0.004)	−0.025 (0.020)
Income quantile 4	−0.023 (0.017)	−0.002 (0.012)	−0.024 (0.024)
Income quantile 5	−0.032† (0.018)	−0.005 (0.033)	−0.050† (0.029)

†*p* < 0.1, *p < 0.05, **p < 0.01, ***p < 0.001. All models control for age, age square, gender, marital status, urban residence, self-rated health, region residence, employment type and occupation. Standard errors are in parentheses.

As shown in Table 3, including certain job-demand measures, such as weekly working hours and on-call duty, significantly reduces education-related differences in depression. Specifically, people with higher education levels tend to work fewer hours, which partly explains these differences. Furthermore, individuals with a 4-year college education or above, the highest level in our study, are less likely to have on-call duties. This helps explain the difference in depression between the highest and lowest education levels. However, the discrete changes in the coefficients for high school and 3-year vocational college education levels are not statistically significant, suggesting that on-call duty does not explain the differences in depression between these groups and the lowest education level. Additionally, the discrete changes in the estimated education coefficients are not statistically significant after controlling for other job-demand measures, such as nightshift frequency and weekend work frequency, indicating that these measures do not account for the education differences in depression.

We also examine the potential mediating effect of job demands in the income-depression association. The inclusion of weekly working hours significantly increases the size of the coefficients of income quantile 2 and income quantile 3, and marginally significantly increases the size of the coefficients of income quantile 5, indicating that the difference in depression between these income levels and the lowest income level is suppressed. In other words, were it not for the longer working hours, the depression difference between people at these higher income levels and people at the lowest income level would have been even greater. The inclusion of on-call duty significantly increases the size of the coefficients of income quantile 3, indicating that the difference in depression between income quantile 3 and the lowest income level is suppressed. In other words, were it not for the higher probability of on-call duty, the difference in depression between the middle and lowest income groups would have been even greater. Similar to their role in the education-depression association, nightshift frequency and weekend work frequency cannot explain the income-depression association.

As shown in Table 4, including job security as a job-resource measure significantly decreases the coefficient for the 4-year college and above group, suggesting that job security may help to explain the depression difference between the highest and lowest education levels. However, job security does not account for depression differences between the lowest and other education levels. Similarly, schedule flexibility may contribute to the depression difference between the 3-year vocational college or 4-year college and above groups and the lowest education level, as indicated by marginally significant coefficient changes, though further investigation is needed. The potential mediating effect of holding a direct supervision position in the education-depression association is unsupported, as coefficient changes across education levels are not statistically significant.

For the income-depression association, including job security and schedule flexibility results in only a marginally significant decrease in the coefficient for income quantile 5. This suggests that job security may help explain the difference in depression between the highest and lowest income groups, although further validation is needed. Similarly, the mediating effect of holding a direct supervisory position in the income-depression association is not supported.

### 3.3 Sensitivity analysis

In the sensitivity analysis, we introduce the logarithm of “yearly net household income” into the baseline model to account for potential confounding effects of household income on the SES-depression association. This analysis is conducted on a reduced sample (N = 6,397) due to missing data on household income. As shown in Table 5, the coefficients for education level and occupational income remain largely unchanged. The log-transformed yearly net household income is negatively significantly associated with depression, indicating that those with higher family income have lower levels of depression. Education and occupational income both show significant associations with depression, even when controlling for each other and other conditions. Overall, the sensitivity analysis supports the robustness of our main findings regarding the SES-depression relationship.

**Table 5:** Regression analysis of depression on socioeconomic status (SES), controlling for household income (*N* = 6,397).

	Model B (Sensitivity analysis)
Education level (Reference = Below high school)	
High school and technical secondary school	−0.317* (0.128)
3-year vocational college	−0.622*** (0.157)
4-year college and above	−0.719*** (0.183)
Occupational income (Reference = Income quantile 1)	
Income quantile 2	−0.489*** (0.144)
Income quantile 3	−0.702*** (0.145)
Income quantile 4	−0.970*** (0.169)
Income quantile 5	−0.683*** (0.175)
Log of yearly net household income	−0.189* (0.074)
Intercept	7.624***
R^2^/Adjusted R^2^	0.101 / 0.098
AIC	37808.810

*p < 0.05, **p < 0.01, ***p < 0.001. All models control for age, age square, gender, marital status, urban residence, self-rated health, region residence, employment type and occupation. Standard errors are in parentheses. AIC stands for Akaike Information Criterion.

## 4 Discussion

Using CFPS 2020 data, a nationally representative sample, we explore the relationships between SES, job conditions, and depression in the Chinese working population. Our first main finding is that both education and income are negatively associated with depression, with education’s association with depression remaining net of income, which is consistent with some Chinese evidence (60) while contradicting some studies from Western contexts, like Schieman and Koltai (11), which suggest that income fully accounts for the association between education and depression. This may stem from China’s cultural emphasis on education, which makes the benefits of education for mental health go well beyond the return on income and more to the self-fulfillment and social recognition that education directly brings (35, 36). Another potential explanation could be the unique relationships that education and income each have with job demands, given that job demands are closely associated with depression. This will be further explored in the subsequent discussion on the mediating role of job conditions in the SES-depression relationship.

Additionally, this study examines the mediating role of job conditions, encompassing job demands and job resources, between SES and depression. Our findings reveal that job demands may act differently in the education and income’s association with depression. As for the mediating role of job demands in the education-depression association, compared to those with less than a high school degree, better educated people have fewer working hours, and those with a 4-year college degree have less likelihood of on-call duty. This contributes to the observed gap in depression between those with lower and higher education levels, suggesting a “stress of lower status” pattern. Regarding the association between income and depression, were it not for the significantly longer working hours required of the second lowest and middle earners and the marginally significantly longer working hours required of the highest earners, as well as the greater likelihood of on-call duty required of the middle earners, the depression gap between these groups of higher earners and the lowest earners would have been even greater, providing partial support to the “stress of higher status” hypothesis. This variation helps clarify the unique relationships that education and income each have with depression. Given that the mediating effects are not consistently significant across all SES levels when compared to the lowest level, these findings should be interpreted with caution. Furthermore, we found no support for a mediating effect of nightshift and weekend shift frequency in either the education-depression or income-depression association.

One possible reason for the lower job demands of the better educated is that higher educational attainment provides an advantage in the employment market, allowing these highly educated individuals to gravitate toward less demanding jobs during the job search phase (61, 62).

Meanwhile, in accord with the existing American evidence (11), our study shows that the income-based differences in depression levels appear to be obscured by some job demands. In our study, higher earners, affected by a sluggish economy and Confucian success values, seem willing to engage in more time-intensive work as a self-motivated trade-off for higher salaries. This self-exploitation benefits the “greedy institutions” but may contribute to the burnout and depression amidst accelerating demands and self-imposed expectations.

Job resources may also mediate the SES-depression association, though the evidence is relatively weak, as most changes in the coefficients for education or income categories after accounting for job resource measures do not reach statistical significance or are only marginally significant. One exception is the mediating effect of job security on the depression difference between the most and least educated groups; specifically, the most educated have higher job security, which may partially explain their lower depression levels compared to the least educated. However, the mediating effect of schedule flexibility in the depression difference between the most and least educated groups is only marginally significant, requiring further investigation to confirm its role. Similarly, higher job security and moderate schedule flexibility among the highest income earners may partially explain their lower depression levels compared to the lowest income earners, but these effects are only marginally significant and warrant further investigation. In addition, we found no support for a mediating effect of holding a direct supervisory role—another job resource measure—in either the education-depression or income-depression association. The preliminary findings highlight the need for societal awareness and action, as poorly educated and low-income workers appear to have fewer job resources, potentially leading to higher levels of depression.

In summary, the association between education and depression persists even when accounting for income can be partly explained by the fact that higher earners are more exposed to some increased job demands, which leads to depression. Conversely, the well-educated generally benefit from less demanding jobs thanks to their advanced level of education.

Our study has several limitations that should be acknowledged. First, we conduct a cross-sectional analysis using the most recent available CFPS data because our primary objective is to examine the distribution of depression across SES and the mediating role of job conditions. This approach helps identify which SES groups are most disadvantaged and the underlying reasons. Additionally, only CFPS 2020 includes certain job demands variables, such as on-call duty, shift work frequency, and schedule flexibility. As a result, we could not use earlier wave data to predict depression outcomes, limiting our ability to perform a longitudinal study. Due to the cross-sectional design, our study does not establish causal relationships. Further longitudinal research is necessary to fully understand the associations between SES and depression. Second, this study does not encompass all typical job demands and resources outlined in the JD-R model. While the JD-R model highlights the importance of interpersonal support from supervisors and colleagues as job resources, and subjective job stress and work–family conflict as key job demands, we were unable to explore their mediating effects due to data limitations. Similarly, for psychological workload, we used measures such as self-reported working hours as its proxies, acknowledging that this approach may be somewhat tenuous. Future research is needed to better align with the JD-R model when more comprehensive Chinese data, including a wider range of job conditions, becomes available. Third, although occupational status is also an important measure of SES, we treat employees’ occupations as a control variable rather than investigating the occupation-depression relationship and how job conditions mitigate this relationship. Given the study’s scope and feasibility, we decided to focus on the association between education, income, and depression. Including occupation as an additional variable of SES could have added complexity beyond our research’s intended scope, which could be investigated in future research.

## 5 Conclusion

The present study sheds light on the SES-depression relationship in the Chinese workforce, revealing the nuanced roles of education and income, and, in particular, highlighting the mediating role of job conditions. This study provides preliminary evidence of distinctive patterns of greater job demands (i.e., working hours and on-call duty) associated with lower education and higher income, and the disadvantaged job resources (i.e., schedule flexibility and job security) associated with lower SES that lead to depression, contributing to the understanding of socioeconomic inequalities in depression in a non-Western context. While the negative associations between SES and depression are robust, the mediating effects of job conditions—particularly job resources—are less conclusive, as the mediating effects of most job resource measures only reach marginal or no statistical significance. Consequently, the findings related to the mediation analysis should be interpreted with caution and further research is needed. Based on these preliminary results, policymakers are encouraged to consider adopting regulations that enhance work-life balance, especially for high earners and less educated individuals, who tend to face higher job demands. Efforts are also needed to reduce the potential disparities in job resources across different SES groups to alleviate the socioeconomic inequalities in depression.

## Data Availability

Publicly available datasets were analyzed in this study. This data can be found at: http://www.isss.pku.edu.cn/cfps/en/data/public/index.htm.

## References

[1] Global Burden of Disease Collaborative Network. (2024). Seattle, United States: Institute for Health Metrics and Evaluation (IHME). Available at: https://vizhub.healthdata.org/gbd-results/ (Accessed November 13, 2024).

[2] Malhi GS Mann JJ. Depression. Lancet. (2018) 392:2299–312. doi: 10.1016/S0140-6736(18)31948-2 30396512

[3] Ren X Yu S Dong W Yin P Xu X Zhou M. Burden of depression in China, 1990–2017: findings from the global burden of disease study 2017. J Affect Disord. (2020) 268:95–101. doi: 10.1016/j.jad.2020.03.011, PMID: 32158012

[4] International Labour Organization. (2024). “ILO modelled estimates and projections database (ILOEST).” ILOSTAT. Available at: https://ilostat.ilo.org/data/ (Accessed November 13, 2024).

[5] WHO. World mental health report: Transforming mental health for all—Executive summary. Geneva, Switzerland: World Health Organization (2022).

[6] Bültmann U Huibers MJH Van Amelsvoort LPGM Kant I Kasl SV Swaen GMH. Psychological distress, fatigue and long-term sickness absence: prospective results from the Maastricht cohort study. J Occup Environ Med. (2005) 47:941–7. doi: 10.1097/01.jom.0000172865.07397.9a, PMID: 16155479

[7] Chisholm D Sweeny K Sheehan P Rasmussen B Smit F Cuijpers P. Scaling-up treatment of depression and anxiety: a global return on investment analysis. Lancet Psychiatry. (2016) 3:415–24. doi: 10.1016/S2215-0366(16)30024-4, PMID: 27083119

[8] Driesen K Jansen NW Van Amelsvoort LG Kant I. The mutual relationship between shift work and depressive complaints – a prospective cohort study. SCAND J Work Env Hea. (2011) 37:402–10. doi: 10.5271/sjweh.3158, PMID: 21526329

[9] Jitender S Tracie OA Katherine AM Gordon JGA. Relationship between household income and mental disorders: findings from a population-based longitudinal study. Arch Gen Psychiatry. (2011) 68:419–27. doi: 10.1001/archgenpsychiatry.2011.15 21464366

[10] Mirowsky J Ross CE. Education, learned effectiveness and health. Lond Rev Educ. (2005) 3:205–20. doi: 10.1080/14748460500372366

[11] Schieman S Koltai J. Discovering pockets of complexity: socioeconomic status, stress exposure, and the nuances of the health gradient. Soc Sci Res. (2017) 63:1–18. doi: 10.1016/j.ssresearch.2016.09.023, PMID: 28202135

[12] Maselko J. Social epidemiology and global mental health: expanding the evidence from high-income to low- and middle-income countries. Curr Epidemiol Rep. (2017) 4:166–73. doi: 10.1007/s40471-017-0107-y, PMID: 28680795 PMC5488107

[13] WHO. (2017). Depression and other common mental disorders. Global Health Estimates. Available at: https://www.who.int/publications/i/item/depression-global-health-estimates (Accessed November 13, 2024).

[14] Zhou G Ding X Zhang W. Minimum wage, income inequality and common prosperity——evidence from CFPS. Inq Econ Issues. (2023) 10:31–47. doi: 10.3773/j.issn.1006-2912.2023.10.31

[15] Jiang R. (2022). “The psychological effects of involution on China’s rising generation,” *2022 5th international conference on humanities education and social sciences (ICHESS 2022)*. 639–645. Atlantis Press.

[16] Zhang K Liu C Ding S. How does working hour affect urban workers’ health? An empirical analysis of the China labor-force dynamic survey. Stud Lab Econ. (2018) 6:107–27.

[17] Bakker AB Demerouti E. The job demands-resources model: state of the art. J Manag Psychol. (2007) 22:309–28. doi: 10.1108/02683940710733115

[18] Cha Y Weeden KA. Overwork and the slow convergence in the gender gap in wages. Am Sociol Rev. (2014) 79:457–84. doi: 10.1177/0003122414528936

[19] Schieman S Whitestone YK Van Gundy K. The nature of work and the stress of higher status. J Health Soc Behav. (2006) 47:242–57. doi: 10.1177/002214650604700304 17066775

[20] Coser LA. Greedy Organisations. Eur Sociol Rev. (1967) 8:196–215. doi: 10.1017/S000397560000151X

[21] Blair-Loy M. Competing devotions: Career and family among women executives. US: Harvard Univ. Press (2005).

[22] Hurtado DA Glymour MM Berkman LF Hashimoto D Reme SE Sorensen G. Schedule control and mental health: the relevance of coworkers’ reports. Community Work Fam. (2015) 18:416–34. doi: 10.1080/13668803.2015.1080663

[23] Baek C Park JB Lee K Jung J. The association between Korean employed workers’ on-call work and health problems, injuries. Ann Occup Environ Me. (2018) 30:19. doi: 10.1186/s40557-018-0225-0 PMC5861623 29581881

[24] Li X Zhang Y. Does the use of robots reduce the workload of workers——evidence from working hours. South China J Econ. (2023) 3:76–84. doi: 10.19592/j.cnki.scje.402206

[25] Xu H Zhou H. Overwork, health loss and income compensation. Stud Lab Econ. (2021) 9:3–26.

[26] Demerouti E Bakker AB. The job demands–resources model: challenges for future research. SA J Ind Psychol. (2011) 37:01–9. doi: 10.4102/sajip.v37i2.974

[27] Gallo LC de los Monteros KE Shivpuri S. Socioeconomic status and health: what is the role of reserve capacity? Curr Dir Psychol Sci. (2009) 18:269–74. doi: 10.1111/j.1467-8721.2009.01650.x, PMID: 22210579 PMC3243949

[28] Schieman S Glavin P. Trouble at the border?: gender, flexibility at work, and the work-home interface. Soc Probl. (2008) 55:590–611. doi: 10.1525/sp.2008.55.4.590

[29] Koltai J Schieman S. Job pressure and SES-contingent buffering: resource reinforcement, substitution, or the stress of higher status? J Health Soc Behav. (2015) 56:180–98. doi: 10.1177/0022146515584151, PMID: 25953278

[30] Mirowsky J Ross CE. Education, personal control, lifestyle and health: a human capital hypothesis. Res Aging. (1998) 20:415–49. doi: 10.1177/0164027598204003

[31] Andersen I Thielen K Nygaard E Diderichsen F. Social inequality in the prevalence of depressive disorders. J Epidemiol Commun H. (2009) 63:575–81. doi: 10.1136/jech.2008.082719 19293167

[32] Inaba A Thoits PA Ueno K Gove WR Evenson RJ Sloan M. Depression in the United States and Japan: gender, marital status, and SES patterns. Soc Sci Med. (2005) 61:2280–92. doi: 10.1016/j.socscimed.2005.07.014, PMID: 16115712

[33] Lorant V. Socioeconomic inequalities in depression: a meta-analysis. Am J Epidemiol. (2003) 157:98–112. doi: 10.1093/aje/kwf182, PMID: 12522017

[34] Schlax J Jünger C Beutel ME Münzel T Pfeiffer N Wild P. Income and education predict elevated depressive symptoms in the general population: results from the Gutenberg health study. BMC Public Health. (2019) 19:430. doi: 10.1186/s12889-019-6730-4, PMID: 31014301 PMC6480596

[35] Cheng M Pan A Shen B. Years of education, class identity and happiness: evidence from the principal-agent theory and CGSS. Sci Decis Mak. (2023) 7:117–32.

[36] Zhang J Xin F. Exploring the changes and causes of higher education yield—an empirical analysis based on CHIP2013, 2018 data. Stat Manag. (2023) 38:34–40. doi: 10.16722/j.issn.1674-537x.2023.06.009

[37] Radloff LS. The CES-D scale: a self-report depression scale for research in the general population. Appl Psychol Meas. (1977) 1:385–401. doi: 10.1177/014662167700100306

[38] Briggs R Carey D O’Halloran AM Kenny RA Kennelly SP. Validation of the 8-item Centre for Epidemiological Studies Depression Scale in a cohort of community-dwelling older people: data from the Irish longitudinal study on ageing (TILDA). Eur Geriatr Med. (2018) 9:121–6. doi: 10.1007/s41999-017-0016-0, PMID: 34654281

[39] Cai Y Kong W Lian Y Jin X. Depressive symptoms among Chinese informal employees in the digital era. Int J Environ Res Public Health. (2021) 18:5211. doi: 10.3390/ijerph18105211 34068883 PMC8156780

[40] Chowhan J Pike K. Workload, work–life interface, stress, job satisfaction and job performance: a job demand–resource model study during COVID-19. Int J Manpow. (2023) 44:653–70. doi: 10.1108/IJM-05-2022-0254

[41] Schaufeli WB Taris TW In: Bauer GF Bridging OH, editors. A critical review of the job demands-resources model: Implications for improving work and health, in bridging occupational, organizational and public health: A transdisciplinary approach. Netherlands: Springer (2014). 43–68.

[42] Yeh H-J. Job demands, job resources, and job satisfaction in East Asia. Soc Indic Res. (2015) 121:47–60. doi: 10.1007/s11205-014-0631-9

[43] Schieman S Glavin P. Education and work-family conflict: explanations, contingencies and mental health consequences. Soc Forces. (2011) 89:1341–62. doi: 10.1093/sf/89.4.1341

[44] Bamberg E Dettmers J Funck H Krähe B Vahle-Hinz T. Effects of on-call work on well-being: results of a daily survey. Appl Psychol-Hlth We. (2012) 4:299–320. doi: 10.1111/j.1758-0854.2012.01075.x, PMID: 23081765

[45] Dettmers J Vahle-Hinz T Bamberg E Friedrich N Keller M. Extended work availability and its relation with start-of-day mood and cortisol. J Occup Health Psychol. (2016) 21:105–18. doi: 10.1037/a0039602, PMID: 26236956

[46] Lee JJ Park E-C Ji H Jang S-I. The effects of on-call work on mental health issues among wage workers in the Republic of Korea. Psychol Health Med. (2020) 25:675–86. doi: 10.1080/13548506.2019.1668565, PMID: 31580728

[47] Shen J Dicker B. The impacts of shiftwork on employees. Int J Hum Resour Manag. (2008) 19:392–405. doi: 10.1080/09585190701799978

[48] Almarzouki AF. Impact of on-call shifts on working memory and the role of burnout, sleep, and mental well-being in trainee physicians. Postgrad Med. (2024) 136:312–7. doi: 10.1080/00325481.2024.2347195, PMID: 38656827

[49] Schieman S Plickert G. How knowledge is power: education and the sense of control. Soc Forces. (2008) 87:153–83. doi: 10.1353/sof.0.0065

[50] Wallace JE Buchanan T. Status differences in interpersonal strain and job resources at work: a mixed methods study of animal health-care providers. Int J Confl Manag. (2019) 31:287–308. doi: 10.1108/IJCMA-08-2019-0135

[51] Cavallari JM Garza JL Ferguson JM Laguerre RA Decker RE Suleiman AO. Working time characteristics and mental health among corrections and transportation workers. Ann Work Expos Heal. (2021) 65:432–45. doi: 10.1093/annweh/wxaa131, PMID: 33604596

[52] Wang ML Narcisse MR Togher K McElfish PA. Job flexibility, job security, and mental health among US working adults. JAMA Netw Open. (2024) 7:e243439. doi: 10.1001/jamanetworkopen.2024.3439, PMID: 38526492 PMC10964112

[53] Pudrovska T Karraker A. Gender, job authority, and depression. J Health Soc Behav. (2014) 55:424–41. doi: 10.1177/0022146514555223 25413803

[54] Schieman S Reid S. Job authority and health: unraveling the competing suppression and explanatory influences. Soc Sci Res. (2009) 69:1616–24. doi: 10.1016/j.socscimed.2009.08.038, PMID: 19800159

[55] Schieman S. Job-related resources and the pressures of working life. Soc Sci Res. (2013) 42:271–82. doi: 10.1016/j.ssresearch.2012.10.003, PMID: 23347475

[56] Rosenfeld RA Van Buren ME Kalleberg AL. Gender differences in supervisory authority: variation among advanced industrialized democracies. Soc Sci Res. (1998) 27:23–49. doi: 10.1006/ssre.1997.0609

[57] Wilson G Mossakowski K. Job authority and perceptions of job security: the nexus by race among men. Am Behav Sci. (2012) 56:1509–24. doi: 10.1177/0002764212458277

[58] Yaish M Stier H. Gender inequality in job authority: a cross-national comparison of 26 countries. Work Occup. (2009) 36:343–66. doi: 10.1177/0730888409349751

[59] Mize TD Doan L Long JS. A general framework for comparing predictions and marginal effects across models. Sociol Methodol. (2019) 49:152–89. doi: 10.1177/0081175019852763

[60] Huang L Sun X Zhou M. Depressive symptoms in Chinese laborers: prevalence and correlated factors among subgroups. J Affect Disord. (2020) 268:141–9. doi: 10.1016/j.jad.2020.03.013, PMID: 32174472

[61] Baum S Ma J Payea K. Education pays 2010: The benefits of higher education for individuals and society. New York, USA: College Board Advocacy & Policy Center (2010).

[62] Liu T Wang D. Education, work experience, and job quality of migrant workers. Popul Res. (2021) 45:85–99.

