# Supplementary material for: Socioeconomic inequalities in depression and the role of job conditions in China
Source: Front Public Health. 2024 Dec 12;12:1464187. doi: 10.3389/fpubh.2024.1464187 (PMC11672342; doi:10.3389/fpubh.2024.1464187)
Supplement: Supplementary file 1 [file Table_1.docx]

Supplementary Material

# Supplementary Tables

| **Table S1A. Job demands regressed on education and income (N = 6,536).** | | | | | |
| --- | --- | --- | --- | --- | --- |
|  | | Weekly working hours | On-call duty | Nightshift frequency | Weekend at work frequency |
| **Education Level** (Reference = Below high school) | | | | | |
| High school and technical secondary school | | -6.428***  (0.579) | -0.011  (0.071) | -0.090  (0.071) | -0.509***  (0.074) |
| 3-year vocational college | | -11.817***  (0.709) | -0.153  (0.088) | -0.289***  (0.088) | -1.011***  (0.087) |
| 4-year college and above | | -12.900***  (0.826) | -0.378***  (0.103) | -0.337***  (0.102) | -1.221***  (0.100) |
| **Occupational Income** (Reference = Income quantile 1) | |  |  |  |  |
| Income quantile 2 |  | 2.988 ***  (0.646) | 0.049  (0.079) | -0.075  (0.082) | 0.119  (0.083) |
| Income quantile 3 |  | 2.895 ***  (0.634) | 0.301***  (0.078) | 0.046  (0.080) | 0.021  (0.079) |
| Income quantile 4 |  | 1.776*  (0.728) | 0.191*  (0.090) | -0.093  (0.091) | -0.151  (0.089) |
| Income quantile 5 |  | 3.437***  (0.717) | 0.180*  (0.089) | 0.107  (0.088) | -0.084  (0.088) |
| **Pseudo R^2^** |  | 0.129 | 0.060 | 0.033 | 0.104 |
| **AIC** |  | 58564.389 | 8786.759 | 11894.390 | 11884.330 |
| Note: * p<0.05, ** p<0.01, *** p<0.001. All models control for age, gender, marital status, urban residence, region residence, employment type and occupation. Standard errors are in parentheses. AIC stands for Akaike Information Criterion. | | | | | |

| **Table S1B. Job resources regressed on education and income (N = 6,536).** | | | | | |
| --- | --- | --- | --- | --- | --- |
|  | | Schedule flexibility | | Direct supervision | Job security |
|  |  | Completely fixed (Reference = Semi-fixed) | Completely flexible (Reference =Semi-fixed) |  | |
| **Education Level** (Reference = Below high school) | | | | | |
| High school and technical secondary school | | -0.292***  (0.080) | -0.650***  (0.110) | 0.464***  (0.110) | 0.056  (0.030) |
| 3-year vocational college | | -0.448***  (0.095) | -0.491***  (0.135) | 0.673***  (0.126) | 0.108**  (0.037) |
| 4-year college and above | | -0.469***  (0.110) | -0.702***  (0.161) | 0.543***  (0.144) | 0.207***  (0.043) |
| **Occupational Income** (Reference = Income quantile 1) | | | | | |
| Income quantile 2 |  | -0.070  (0.091) | -0.539***  (0.123) | 0.048  (0.148) | -0.037  (0.033) |
| Income quantile 3 |  | -0.095  (0.088) | -0.755***  (0.122) | 0.388**  (0.133) | 0.040  (0.033) |
| Income quantile 4 |  | -0.254*  (0.099) | -0.613***  (0.136) | 0.721***  (0.141) | 0.031  (0.038) |
| Income quantile 5 |  | -0.492***  (0.097) | -0.504***  (0.130) | 1.300***  (0.133) | 0.094*  (0.037) |
| **Pseudo R^2^** |  | 0.049 | | 0.187 | 0.086 |
| **AIC** |  | 13055.510 | | 5294.989 | 19878.847 |
| Note: * p<0.05, ** p<0.01, *** p<0.001. All models control for age, gender, marital status, urban residence, region residence, employment type and occupation. Standard errors are in parentheses. AIC stands for Akaike Information Criterion. | | | | | |

| **Table S2. Depression regressed on SES and job conditions (N=6,536).** | | | | | | | | | | | |
| --- | --- | --- | --- | --- | --- | --- | --- | --- | --- | --- | --- |
|  | **Model S1** | **Model S2** | **Model S3** | **Model S4** | **Model S5** | **Model S6** | **Model S7** | **Model S8** | **Model S9** | **Model S10** |  |
| **Education Level** (Reference= Below high school) | | | | | | | | | | | |
| High school and technical secondary school | -0.235  (0.128) | -0.315*  (0.126) | -0.306*  (0.126) | -0.336**  (0.127) | -0.280*  (0.127) | -0.289*  (0.127) | -0.313*  (0.127) | -0.297*  (0.126) | -0.279*  (0.126) | -0.263*  (0.127) |  |
| 3-year vocational college | -0.476***  (0.157) | -0.605***  (0.154) | -0.594***  (0.154) | -0.625***  (0.156) | -0.517**  (0.157) | -0.593***  (0.155) | -0.620***  (0.155) | -0.582***  (0.154) | -0.557***  (0.154) | -0.486**  (0.157) |  |
| 4-year college and above | -0.592***  (0.183) | -0.707***  (0.179) | -0.717***  (0.180) | -0.749***  (0.182) | -0.616**  (0.183) | -0.716***  (0.180) | -0.750***  (0.180) | -0.667***  (0.179) | -0.638***  (0.180) | -0.549**  (0.183) |  |
| **Occupational Income** (Reference = Income quantile 1) | | | | | | | | | | | |
| Income quantile 2 | -0.562***  (0.141) | -0.529***  (0.141) | -0.517***  (0.141) | -0.533***  (0.141) | -0.552***  (0.141) | -0.511***  (0.142) | -0.526***  (0.141) | -0.533***  (0.141) | -0.524***  (0.141) | -0.549***  (0.141) |  |
| Income quantile 3 | -0.822***  (0.139) | -0.821***  (0.139) | -0.792***  (0.139) | -0.799***  (0.139) | -0.857***  (0.139) | -0.769***  (0.140) | -0.787***  (0.139) | -0.762***  (0.139) | -0.751***  (0.139) | -0.819***  (0.139) |  |
| Income quantile 4 | -1.137***  (0.160) | -1.135***  (0.160) | -1.104***  (0.160) | -1.123***  (0.160) | -1.155***  (0.160) | -1.093***  (0.160) | -1.114***  (0.160) | -1.092***  (0.159) | -1.074***  (0.160) | -1.117***  (0.159) |  |
| Income quantile 5 | -0.941***  (0.158) | -0.917***  (0.158) | -0.911***  (0.158) | -0.905***  (0.158) | -0.967***  (0.158) | -0.867***  (0.158) | -0.894***  (0.159) | -0.849***  (0.157) | -0.821***  (0.159) | -0.886***  (0.159) |  |
| **Job Demands** | | | | | | | | | | | |
| Weekly working hours | 0.012***  (0.003) |  |  |  | 0.011***  (0.003) |  |  |  |  | 0.010**  (0.003) |  |
| On call (On call=1) |  | 0.527***  (0.092) |  |  | 0.457***  (0.094) |  |  |  |  | 0.428***  (0.094) |  |
| Nightshift: No more than once a week |  |  | 0.413***  (0.118) |  | 0.302*  (0.120) |  |  |  |  | 0.243*  (0.131) |  |
| Nightshift: Several times a week/every day |  |  | 0.565***  (0.127) |  | 0.412**  (0.131) |  |  |  |  | 0.354**  (0.131) |  |
| Weekend at work: Several times a month but less than every week |  |  |  | 0.531***  (0.143) | 0.308*  (0.146) |  |  |  |  | 0.299*  (0.145) |  |
| Weekend at work: Every week |  |  |  | 0.157  (0.116) | -0.161  (0.124) |  |  |  |  | -0.228  (0.124) |  |
| **Job Resources** | | | | | | | | | | | |
| Schedule flexibility: Completely fixed (Reference =Semi-fixed)  Schedule flexibility: Completely flexible (Reference =Semi-fixed) |  |  |  |  |  | 0.283***  (0.101) |  |  | 0.271**  (0.101) | 0.231*  (0.101) |  |
|  |  |  |  |  |  | 0.352***  (0.142) |  |  | 0.257  (0.142) | 0.196  (0.142) |  |
| Direct supervision (Supervisor=1) |  |  |  |  |  |  | -0.035  (0.135) |  | 0.001  (0.134) | -0.034  (0.134) |  |
| Job security |  |  |  |  |  |  |  | -0.403***  (0.052) | -0.398***  (0.052) | -0.203**  (0.053) |  |
| **Intercept** | 6.778***  (0.745) | 7.334***  (0.728) | 7.208***  (0.730) | 7.354***  (0.735) | 6.641***  (0.744) | 7.336***  (0.731) | 7.506***  (0.729) | 9.211***  (0.758) | 9.004***  (0.761) | 8.257***  (0.783) |  |
| **R^2^/Adjusted R^2^** | 0.104 / 0.103 | 0.108 / 0.104 | 0.107 / 0.103 | 0.105 / 0.101 | 0.105 / 0.101 | 0.104 / 0.101 | 0.103 / 0.100 | 0.111 / 0.108 | 0.112 / 0.109 | 0.121 / 0.117 |  |
| **AIC** | 38605.938 | 38594.563 | 38602.887 | 38614.933 | 38614.933 | 38619.497 | 38627.144 | 38566.725 | 38564.987 | 38512.337 |  |
| Notes: * p<0.05 ** p<0.01 *** p<0.001. All models control for age, age square, gender, marital status, urban residence, self-rated health, region residence, employment type and occupation. Standard errors are in parentheses. AIC stands for Akaike Information Criterion. | | | | | | | | | | | |
